# Caregivers’ health-seeking behaviour for children participating in an integrated school health programme in KwaZulu-Natal, South Africa

**DOI:** 10.4102/phcfm.v15i1.3822

**Published:** 2023-02-17

**Authors:** Gbotemi B. Babatunde, Olagoke Akintola

**Affiliations:** 1 School of Public Health, Faculty of Community and Health Sciences, University of the Western Cape, Cape Town, South Africa

**Keywords:** access, caregivers, school-going children, school-based healthcare services, school health programme, integrated school health policy, low-resource communities, South Africa

## Abstract

**Background:**

Caregivers are active members of the healthcare team, and the uniqueness of their role in caring for a sick child is holistic, as no other healthcare team member is consistently aware of all the facets of the child’s life. The integrated school health programme (ISHP) aims to improve access to healthcare services and promote equity for school-going children by delivering comprehensive healthcare services. However, not much attention has been paid to understanding caregivers’ health-seeking experiences within the context of the ISHP.

**Aim:**

This study sought to understand caregivers’ health-seeking behaviour for their children participating in the ISHP.

**Setting:**

Three low-resource communities were chosen within the eThekwini District of the KwaZulu-Natal province, South Africa.

**Methods:**

This study utilised a qualitative research design. We recruited 17 caregivers using purposive sampling. Semistructured interviews were conducted, and the data were analysed using thematic analysis.

**Results:**

Caregivers explored multiple means of care, ranging from managing the children’s health conditions based on previous experiences to visiting traditional healers and administering traditional medicines. Caregivers delayed health seeking due to low literacy levels and financial barriers.

**Conclusion:**

Although ISHP has expanded its coverage and the range of services provided, the study suggests the need to implement interventions focused on providing support to caregivers of sick children within the ISHP context.

**Contribution:**

The findings of the study highlight the need to develop potential schemes to address transportation barriers to accessing healthcare services for school-going children.

## Introduction

Children in sub-Saharan Africa (SSA) are predisposed to several risk factors, including poverty, poor nutrition, a lack of adequate care and support and immediate risk in the community that impact their health and educational outcomes.^1,2^ As a result, child mortality remains a significant public health issue in SSA, where one in 12 children under 5 years dies because of preventable or treatable illnesses such as malaria, diarrhoeal diseases, measles, respiratory infections, human immunodeficiency virus (HIV), tuberculosis, pneumonia and malnutrition.^3,4^ In addition, previous studies have established that inequalities in access and poor utilisation of healthcare services result in poor health outcomes in children.^2,5^ These associations are strengthened by different family- and community-level factors, such as poor health-seeking practices because of low literacy levels. Other factors may include stigmatisation, poverty, unemployment, homelessness, younger maternal age and health system–level factors such as the distance to healthcare facilities, cost of healthcare services, availability of quality services and attitudes of healthcare workers.^2^

In South Africa, a significant amount of reported child mortality results from severe illnesses, which can be prevented by prompt and appropriate health-seeking.^6^ Children depend on their caregivers to attend to their health needs, as they do not have the independence or means to seek professional healthcare directly.^7^ Hence, caregivers are active members of the healthcare team, and the uniqueness of their roles in caring for a sick child is holistic, as no other healthcare team member is consistently aware of all the facets of the child’s life.^8,9^ Therefore, the effective management of a child’s health condition requires the caregivers’ timely recognition of the health problem, the decision to seek help and the appropriate utilisation of healthcare services.^10^ However, decision-making on where and when to seek help is a complex process often influenced by the affordability, availability and proximity of healthcare services.^11^

Thurston et al.^12^ described three stages of health-seeking behaviour: (1) problem recognition – when an individual becomes aware of the health condition or symptoms; (2) decision to seek help – when an individual decides to seek healthcare services from professionals or nonprofessionals; (3) service selection and utilisation – when an individual decides to seek healthcare services from particular care providers.^13^ However, whether caregivers go through these stages consecutively is unclear.^14^ Caregivers, especially those residing in low-resource communities, explore several means of treatment, including traditional and biomedical healthcare, and those with limited access to biomedical care resort to traditional treatments.^15^ Medical pluralism is a common practice in low-resource communities. In most societies, people seek diagnosis and treatment from different sources ranging from self-care (home remedies), traditional healers (herbalists or spiritual healers), paraprofessionals (medical assistants) to medical professionals (nurses, doctors and other specialists), either at primary health centres or at hospitals.^16^ Some caregivers never begin the process because they cannot recognise the problem,^17^ while others may experience some barriers because of the stress associated with health-seeking.^18^

One of the initiatives jointly designed by the Departments of Basic Education and Health and Social Development in 2012 to improve access to healthcare services and promote equity for school-going children is the Integrated School Health Policy (ISHP).^19^ The ISHP team, which comprises professional and enrolled nurses, delivers comprehensive healthcare services ranging from screening to referral to meet the health needs of school-going children.^20^ The programme is being implemented across nine provinces, starting with schools in the most disadvantaged communities. The services provided include: (1) screening and assessment; (2) on-site services such as deworming, immunisation and treatment of minor ailments and provision of health education; and (3) referral and follow-up services.^19^ Schools are no longer viewed as just learning centres but also supportive centres for providing essential health service packages, nutritious meals, a healthy physical environment with safe water and sanitation and skill-based health education.^20^ The school year provides an ideal opportunity for health education and interventions, as most children spend close to 13 formative years in school. Therefore, school health programmes are the most effective way of ensuring children’s well-being, when adequately implemented.^19^

The ISHP policy document highlighted the significance of actively engaging with caregivers to promote appropriate health-seeking practices. It stated that the school health nurses should build relationships with the learners’ caregivers, provide adequate information about the school healthcare services and educate them about the children’s health conditions.^20^ However, not much attention has been paid to understanding caregivers’ health-seeking experiences within the context of the ISHP. Therefore, this study is pertinent as it explores caregivers’ health-seeking behaviours before and after their children have received care through the ISHP, which involves exploring the relationship between the caregivers and the health nurses. In addition, it sought to understand how these relationships influence caregivers’ health-seeking behaviour for their school-going children.

## Research methods and design

### Study design

This study utilised an exploratory and descriptive qualitative design, using in-depth interviews to generate information about caregivers’ health-seeking behaviour for their school-going children.^21^ The design allowed the authors to capture the lived experiences of caregivers and health-seeking behaviour for their services provided through the ISHP.^22^

### Study setting

Three low-resource communities were chosen within the eThekwini District of the KwaZulu-Natal province, South Africa. These peri-urban communities are characterised by poverty because of high unemployment rates and a low skills base. According to Colin and Charlton,^23^ the unemployment rate in peri-urban communities in Durban is about 40%. Furthermore, about 25% of those employed earn less than R500.00 ($30.00) monthly. Schools within the communities fall within the Quintiles 1–3 categories: schools situated in the most economically disadvantaged communities.^24^

### Study population and sampling

A nonprobability purposive sampling strategy was used to recruit 17 participants who had the potential to provide in-depth information because of their previous experiences and availability to participate in the study.^25^ The researchers developed a set of selection criteria to recruit study participants.^26^ The selection criteria were as follows: a caregiver must be responsible for at least one primary school-going child (Grade R – Grade 7) and be resident in one of the peri-urban communities where the study was conducted. Researchers sought to include participants with various experiences relating to the research topic.^22,26^ Participants belonging to two categories: (1) caregivers whose children were screened and treated on-site (in school) and (2) caregivers whose children were screened and referred for further assessment and treatment, were therfore recruited. The latter category comprised two subcategories: (1) caregivers who followed up by seeking treatment for their children and (2) caregivers who did not seek treatment for their children. Community entry was facilitated by a nonprofit organisation (NPO) that collaborated with the health and education departments to implement the school health initiative in the communities. The NPO provided school health services through trained school health nurses. The school health nurses working with this NPO assisted with the selection of participants. In order to identify participants, researchers leveraged the existing relationship between the school health nurses and the caregivers whose children were participating in the ISHP. The school health nurses, telephonically and during home visits, approached caregivers to participate in the study. The nurses also assisted with scheduling a convenient time for the interviews. Before the interviews, the nurses introduced the research team to the participants.

### Data collection

A semistructured interview schedule containing open-ended questions was developed based on relevant literature on health-seeking behaviour and access to healthcare services. It covered a range of themes, including demographic information about the caregivers and their children and the caregivers’ health-seeking behaviour for their school-going children.

Three research assistants (two women and a man) who were postgraduate students and were proficient in both isiZulu and English were recruited and trained by one of the authors (G.B.B.) to help with data collection. The authors provided the research assistants with detailed information about the study context, settings and participants. The interview schedule, initially constructed in English, was jointly translated to isiZulu by the research assistants during one of the meetings to ensure accurate translation and uniformity in the research procedure. The interview schedule was back-translated into English. In addition, the research assistants piloted the interview guide and revised some of the questions. This was to ensure the translated questions were accurately phrased. For example, the question on cultural beliefs was rephrased to capture the participants’ broad views about different health conditions. The research assistants at the participants’ homes conducted the interviews in isiZulu. Each interview lasted between 30 min and 45 min and was audio-recorded. G.B.B. was present to take notes during the interview sessions. Data collection occurred between April 2016 and June 2016.

### Data analysis

All the interviews were transcribed verbatim, translated and back-translated where required. The transcribed data were analysed following the five steps recommended by Braun and Clark^27^ for thematic analysis. To ensure rigour and establish the credibility of the data, all the transcripts were thoroughly reviewed. Similarities within and across all the transcripts were identified, and the data were meaningfully clustered under the appropriate themes. The field notes further helped in analysing the data and understanding the themes. G.B.B. coded the data set and developed themes around the phenomena of interest until saturation was reached and no new themes emerged from any of the transcripts. The themes were extracted, and a matrix was created using Excel software (Microsoft Corporation, Redmond, Washington, United States). O.A. reviewed the matrix to ensure the analysis focused on exploring caregivers’ health-seeking behaviours and experiences. Both researchers reached an agreement after extensive discussions on the themes.

### Ethical considerations

Ethical approval was provided by the Human Social Sciences Research Ethics Committee of the University of KwaZulu-Natal (reference number HSS/0570/016). All the participants provided consent to participate and for the interviews to be audio-recorded.

## Results

Caregivers’ sociodemographic characteristics are presented in Table 1. An overwhelming majority (*n* = 16) of the total participants (*n* = 17) were female. The participants’ ages ranged from 27 to 70 years, and most caregivers were between 27 and 32 years. Most participants were the children’s mothers (*n* = 8), followed by grandmothers (*n* = 5). Most participants were unemployed (*n* = 7), only a few (*n* = 4) were employed and others were either self-employed, pensioners or receiving government grants. The study participants provided care for 24 children attending primary schools. A slightly larger number of the children (*n* = 13) were female, and the ages of the children ranged from 5 to 15 years.

**TABLE 1:** Participants’ characteristics (*N* = 17).

Characteristics	Frequency	Percentage
**Gender**		
Male	1	5.88
Female	16	94.12
**Age**		
15–20	0	0.00
21–26	0	0.00
27–32	5	29.35
33–38	4	23.53
39–44	1	5.90
45–50	2	11.76
51–56	2	11.76
57–62	1	5.90
63–68	1	5.90
69–74	1	5.90
**Relationship with children**		
Mother	8	47.05
Grandmother	5	35.31
Aunt	2	11.76
Stepmother	1	5.88
Father	1	5.88
**Employment status**		
Unemployed	7	41.18
Employed	4	23.53
Self-employed	3	17.65
Grant receiver	2	11.76
Pensioner	1	5.88

The health conditions mentioned were also grouped into major and minor ailments, as shown in Table 2.

**TABLE 2:** Children’s health conditions.

Health conditions	Frequency (*N*)	Percentage
**Major ailments**		
Skin conditions (rash, scabies and sores)	8	26.7
Eye problems	6	20.0
Ear problems (wax impaction, infection and hearing loss)	4	13.3
Teeth extraction	3	10.0
Asthma	2	6.7
HIV	2	6.7
Epilepsy	1	3.3
**Minor ailments**		
Stomach ache or worms	2	6.7
Headache or fever	1	3.3
Cold or flu	1	3.3
Amenorrhea or menstrual pain	1	3.3

HIV, human immunodeficiency virus.

Note: The frequency (*N*) will not add up to the total number of children because some children were diagnosed with more than one condition. It is important to note that some children were diagnosed and treated for more than one health condition on-site. Sometimes, the school health nurses gave the children medications to take home.

## Themes and subthemes

**Figure 1:**
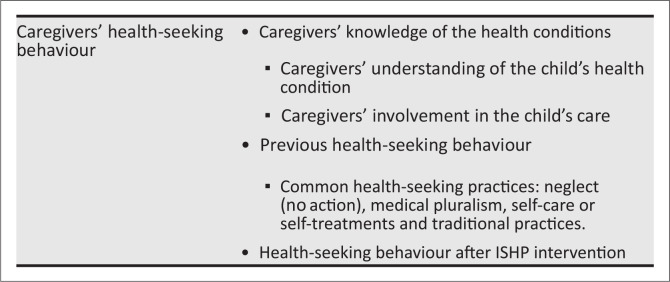
Themes and subthemes.

The results have been grouped into subthemes representing the factors influencing caregivers’ health-seeking behaviour. They include caregivers’ knowledge of their children’s health conditions, the identification of the health conditions and the caregivers’ previous and present health-seeking behaviour. The themes and subthemes are presented in Figure 1.

### Caregivers’ knowledge of the health condition(s)

Most caregivers mentioned that they were unaware that their children had a health condition and would not have noticed the problem if they had not been screened at school. The participants noted that the school health nurses identified most health conditions during the routine health assessment process.

‘The health condition was first noticed in school. I didn’t know he was suffering from an eye problem before I got the letter.’ (Caregiver G, Primary school C, Female)

However, some caregivers mentioned that they were aware of their children’s health conditions before the school informed them. These caregivers noticed the symptoms but could not ascertain the problem and could not take any steps to seek the appropriate healthcare services until the children were screened in school. This was the case with a mother whose child had an eye problem:

‘I saw the signs before the school informed me about the eye problem. But I didn’t know what it was before the school sent the letter.’ (Caregiver C, Primary school B, Female)

Some children complained about their health conditions to their caregivers before being screened and treated in school. However, the caregivers mentioned that they didn’t realise the severity of the health condition until the school informed them about their children’s health condition. A grandmother whose grandchild had a tooth problem said:

‘The child complained about the problem to me before she was screened in school. I thought the tooth would come out itself until the nurses sent a letter informing us that she would be taken to the hospital.’ (Caregiver M, Primary school D, Female)

Sometimes, caregivers or neighbours noticed the children’s health condition. They could recognise the health conditions and the severity but still couldn’t decide whether to seek healthcare services for the children or not. For example, a mother of a child with skin rashes mentioned that:

‘I [*the mother*] and my neighbour saw the rash before the school sent a letter to us. I knew skin rash is a serious health condition.’ (Caregiver D, Primary school A, Female)

#### Caregivers’ understanding of children’s health conditions

Most caregivers demonstrated a fair understanding of their children’s health conditions. Those who identified the health condition(s) described the symptoms the children presented before they were screened in school. Although some caregivers had no idea what the symptoms were when they initially noticed that their children had a health challenge, other caregivers could recognise the health problem because of their previous experiences. A caregiver whose grandchild had a knee tumour said:

‘He started limping and limping, but as time passed, we realised that the swollen knee was not deflating. His mother also had it when she was younger, it is called tumour.’ (Caregiver E, Primary school A, Female)

Some caregivers who could recognise the health problem and attempted to treat or manage the conditions, but these weren’t sufficient to alleviate the respective health conditions. A caregiver whose child had a skin rash said:

‘I knew it was an infection, I think, two months ago. It was like he was burning. I know it is contagious, and the things of the affected person must be kept away from others, so I was careful with his clothes. I washed them separately because I didn’t want the other children to be infected. He also stayed at home for a while because people with rashes need to be kept away from others until they are healed. I have seen it on my neighbour’s children, so I know it’s contagious.’ (Caregiver J, Primary school D, Female)

#### Caregivers’ involvement in children’s care

Most of the caregivers were actively involved in their children’s healthcare. They mentioned that their decision to seek help for the child was influenced by the health conditions’ impact on the children. These include pains, discomfort, academic setback, absenteeism which results in poor academic performance, isolation from other children and behavioural disorders. A mother whose daughter had a severe eye defect mentioned that:

‘She is not supposed to be in her present grade, her mates are in high school now. She started school early, but because she couldn’t see the board clearly like other children, she had to repeat some grades.’ (Caregiver C, Primary school B, Female)

Another caregiver whose child had a psychological disorder stated:

‘He probably would have completed primary school if not for his illness [*epilepsy*]. He stayed at home without learning for about three years because the fits were regular. And when something upsets him, he just gets angry, even here at home, when he starts, we can’t control him we have to get the neighbours to assist us.’ (Caregivers I, Primary school A, Female)

Most of the caregivers were concerned about their children’s state of health. The majority of the caregivers described the psychological impacts the children’s health condition(s) have on them. The psychological impacts mentioned are depression, helplessness, dissatisfaction, confusion and exhaustion. These psychological impacts are aggravated by the caregivers’ inability to access the appropriate healthcare services. These are some of the caregivers’ expressions:

‘I’m worried about him, but I’m tired of complaining about his injuries.’ (Caregiver O, Primary school D, Female).

‘I’m not happy about their health condition, but I’m helpless at the moment.’ (Caregiver P, Primary school D, Female)

The caregiver whose child had a psychological disorder reported that the child sometimes misbehaves because of his health challenge. She narrated a scenario when the child fought a teacher in the school, and she was invited to the school by the principal. She said:

‘I had to apologise to the teacher on his behalf, and you know we were in a room full of teachers, and my heart was so sore. I just became emotional and began crying and walked out.’ (Caregiver I, Primary school A, Female)

### Previous health-seeking behaviour

#### Common health-seeking practices

Caregivers who were aware of their children’s health conditions before the intervention of the school health programme made different attempts to improve the child’s health condition. They engaged in other health-seeking practices, ranging from self-care to seeking treatment at the health facilities closest to them. Some mentioned that they used the knowledge gained from their previous experiences in treating the children. Others sought advice from their friends and neighbours. They took the children to clinics close to them and used *muthi* (traditional medicine) from traditional healers. A caregiver stated that her reason for exploring both means was that she wanted the child to recover quickly. They also mentioned that they used other forms of treatment, for example, warm salt water to swab a child’s knee tumour and herbal ointment to treat skin rashes and so on. A caregiver of a child with skin rashes said:

‘I took the child to a traditional healer, who gave us some traditional medications and ointment. I would sometimes use the herbal ointment on her skin. I will also give her some traditional medicine to drink, wash her clothes separately and spread them outside to dry. I later took her to the clinic. I used everything I could lay my hands on, I used both because I wanted her to be healed quickly.’ (Caregiver D, Primary school A, Female)

Other caregivers, however, mentioned that they tried several traditional medications. Still, there was no improvement in the children’s health condition until they were screened, treated and referred for further treatment by the school health nurses. A caregiver whose child attended Primary school B narrated his experience of treating the child’s skin condition with various traditional herbs before the child was referred to the Community A primary health care centre by the school health nurses:

‘We tried taking the child to a traditional healer, which didn’t work, but we did not give up because it needed to be conquered. I actually tried the natural herbs, but they didn’t help. I tried *umdoni* and *umgadankawu* [*names of herbs*], and they didn’t work … we only started seeing improvements when he started taking the medications from the clinic.’ (Caregiver A, Primary school B, Male)

Caregivers who were not aware of the child’s health condition did not attempt to seek healthcare services for the children. Some mentioned that they recognised and treated the children’s symptoms of other health conditions. However, they could not recognise the presence of the major ailments identified by the school health team. This could be because a comprehensive medical assessment procedure was needed to identify those health conditions. A good example of these health conditions is eye defects. A caregiver stated that:

‘Each time she complained of anything [*headache, flu or fever*], I usually take her to the clinic, and she gets treated. But concerning her eye problem, I didn’t do anything because I was unaware.’ (Caregiver C, Primary school B, Female)

A few caregivers also indicated that they did not seek healthcare for the children because they thought the conditions would resolve naturally. For example, a caregiver who is responsible for eight grandchildren, whose ages ranged between 5 and 13 years, stated her reasons for not accessing healthcare services for five of the children before they were screened and treated by the school health nurses:

‘No, I didn’t try anything. I don’t want to lie. I haven’t tried anything because the child with teeth problem is still developing new teeth, so I thought the teeth would go out. I tried applying *muthi* [*traditional medication*] on the one with skin rash. I just told the one with cold/flu to cover herself properly.’ (Caregiver M, Primary school D, Female)

Even though the caregivers explored several treatment options, they did not associate the children’s health conditions with their traditional or cultural beliefs. This suggests that caregivers are aware of the biomedical health risk factors related to their children’s health conditions. A caregiver whose son was diagnosed with an eye defect mentioned that:

‘I don’t think his eye problem is related to our tradition or any ritual. I also don’t think traditional treatments will be better than going to the clinic.’ (Caregiver G, Primary school C, Female)

### Current health-seeking behaviour

After the children were screened in school, some were treated on-site, while others were referred to public health facilities for further treatment. The majority of the caregivers of the school-going children referred for further treatment took their children to the referral sites to seek healthcare services for the children.

‘I later took her to the hospital after she was referred by the school nurse, and she is fine now.’ (Caregiver B, Primary school B, Female)

‘I took him to the clinic the day after I was called.’ (Caregiver H, Primary school C, Female)

However, some caregivers did not follow the advice to take their children for further healthcare interventions. Two grandmothers stated that they care for many children and are not strong enough to go through the stress of taking them to referral sites. One of the grandmothers said that taking the child to the hospital is the mother’s responsibility. Although the child lives with her and takes care of her other needs, she did not want to over-extend herself to take the child to the hospital. She said:

‘I can’t go through that anymore. I did it for his mother and her siblings. It’s his mother’s responsibility … she must come and take him to the hospital. I’m no longer strong to go through that stress.’ (Caregiver O, Primary school D, Female)

Another grandmother complained about the challenge of taking her many children for treatments, given her illnesses:

‘I have eight of them living with me, it’s sometimes too much for only me to handle. I don’t have the time, and my health is not good enough. They referred the ones with eye, ear and teeth problems and the one with HIV to the hospital, but I haven’t taken them.’ (Caregiver I, Primary school A, Female)

Financial barriers and time constraints are the most common reasons mentioned by caregivers for not taking their school-going children for healthcare services. The grandmother with eight children mentioned that she is not financially capable and needed to stay at home to attend to her business, which is the major source of income for the family:

‘I don’t have the time to take them. I have to sell ice cream at home to feed them, that is where I get money. I also don’t have money for transportation.’ (Caregiver M, Primary school D, Female)

Another caregiver said he is waiting for his salary before taking the child for medical treatment:

‘The only problem we had was money. I don’t have money. I have been waiting to get paid and then take him to the hospital.’ (Caregiver A, Primary school B, Male)

While children with minor ailments were treated in school, some children with major ailments, especially those with eye and teeth problems, were taken to the hospital for further treatment by the nurses or the mobile clinic. Some of the caregivers mentioned that:

‘The mobile clinic took the child with the eye problem to the hospital. She was given medications and also promised to give her glasses. The child with flu was also given some medication from school.’ (Caregiver M, Primary school D, Female)

## Discussion

This study reveals that the burden of caring for a sick child falls largely on mothers and grandmothers. Also, the findings in this study show that identifying health conditions and deciding when and where to seek healthcare is a complex process. Moreover, caring for sick children results in new responsibilities associated with financial, physical and psychological distress. These findings support previous studies conducted in low-resource settings which show that mothers and grandmothers are the most active participants in the child healthcare process, and the various responsibilities associated with caring for a sick child fall on them.^6,28,29^

The authors found that efforts to provide care for a child hinge on the caregivers’ ability to identify the condition, financial capability, access to healthcare services and willingness to seek healthcare. The findings further revealed that the participants’ decision-making on the appropriate pathway to care for a sick child is influenced by their previous experiences, the conditions’ perceived severity and the ISHP intervention. For example, caregivers, mostly grandmothers who had previously treated a particular condition, could easily identify the symptoms, determine the severity and seek adequate care. This finding aligns with the findings of Haskins et al.,^6^ which stated that grandmothers are key decision-makers in children’s healthcare because of their knowledge and experience. In contrast, other caregivers without previous knowledge or experience only sought help after the school health nurses intervened. This underscores the significance of routine screening, part of the school health services provided through the ISHP.

Health literacy levels determine a caregiver’s health-seeking behaviour. Health literacy is the ability to understand basic health information and make the right health-seeking decisions to promote health and well-being. Previous studies among different populations globally have revealed that low health literacy is related to late identification, poor use of healthcare resources, poor ability to interpret health messages and poor health outcomes.^30,31^ Some caregivers in this study would not have accessed care for their children if the conditions were not identified in school and provided with care through the ISHP. This highlights the value of the school health programme as an effective medium to educate caregivers about their children’s health and improve their health literacy levels. A previous study by May et al.^32^ noted that caregivers’ level of health literacy–related caregiving skills predicts their health-seeking behaviours and disposition when caring for children with mild acute conditions. According to May et al.^32,^ equipping caregivers with the skills required to identify health conditions, determine the severity of the conditions and navigate the healthcare system will improve children’s health outcomes. This study shows that the information provided by the school health nurses about the children’s health condition motivates the caregivers to access quality healthcare services for their children. This opportunity provided through the relationships between the school health nurses and the caregivers could be maximised to enhance caregivers’ health literacy levels.

The caregivers participating in this study did not attribute the causes of their school-going children’s health challenges to religious and traditional beliefs. They also did not perceive traditional treatment to be better than medical treatment. This finding could be because the study was conducted after the school health intervention, which might have influenced the caregivers’ current health-seeking practices and their perceptions of the children’s illnesses. Nevertheless, some caregivers visited traditional healers and used traditional treatments alongside biomedical treatment. These findings contrast the findings of Haskins et al.^6^, in which the authors found that caregivers attributed the causes of illnesses to religious and cultural beliefs and had a high level of confidence in traditional medicine. Other direct and indirect means of health promotion and education in the communities could also account for this contradiction.

Most caregivers were unemployed, and the few employed earned low incomes. This lack of income influenced caregivers’ decision to seek healthcare services for their children. Before the intervention of the school health programme, caregivers utilised cheap and easily accessible alternatives such as traditional medicines (*muthi*), homemade remedies and other natural treatments. Also, caregivers of children referred for further treatment mentioned they delayed health-seeking because of the cost of transportation and the stress associated with navigating services at the referral sites. This supports a previous study in South Africa that posits that people living in rural communities are more likely to delay accessing healthcare because of financial constraints.^33^ Although the caregivers of children who received healthcare in school stated that the healthcare services provided through the ISHP relieve the financial burden associated with caring for their sick children, transport remains a major barrier to obtaining prompt and quality care for school-going children.

Delays in seeking healthcare services for school-going children could exacerbate the children’s health conditions, negatively affecting school attendance and learning capability, which undermines the effort of ISHP. This finding indicates that policymakers need to develop possible strategies to address this transportation barrier to access. This could be in the form of providing financial assistance towards the cost of transportation or providing vehicles to transport the children to the health facilities. It is essential to revisit the section of the ISHP that states that the Department of Social Development (DSD) will be responsible for providing transportation support to referral sites.^20^

The study limitations should be considered when interpreting the findings of this study. Different contextual factors may affect the transferability of the findings of this study, as the study was localised to three low-resource communities in KwaZulu-Natal.

## Conclusion

Caregivers’ health-seeking behaviour determines the health and educational outcomes of school-going children. Hence, it is important to promote appropriate healthcare-seeking for children in low-resource communities. The findings in this study revealed that ISHP provides the opportunity to educate and encourage caregivers to be actively involved in the care process. Efforts should be made to develop and implement interventions focused on providing support to caregivers of sick children within the ISHP context. It is important to provide transportation support and increase home visits to facilitate active engagement between the caregivers and the school health team. This could help improve the effectiveness of the ISHP and also help in achieving its goal, which is to improve the educational and health outcomes of school-going children.
